# Benzimidazole and aminoalcohol derivatives show in vitro anthelmintic activity against *Trichuris muris* and *Heligmosomoides polygyrus*

**DOI:** 10.1186/s13071-022-05347-y

**Published:** 2022-07-08

**Authors:** Elora Valderas-García, Cécile Häberli, María Álvarez-Bardón, Nerea Escala, Verónica Castilla-Gómez de Agüero, Jennifer de la Vega, Esther del Olmo, Rafael Balaña-Fouce, Jennifer Keiser, María Martínez-Valladares

**Affiliations:** 1 Instituto de Ganadería de Montaña, CSIC-Universidad de León, 24346 Grulleros, León, Spain; 2 Departamento de Ciencias Biomédicas, Facultad de Veterinaria, Universidad de León, 24071 León, Spain; 3 Swiss Tropical and Public Health Institute, Socinstrasse 57, 4051 Basel, Switzerland; 4 University of Basel, 4003 Basel, Switzerland; 5 Departamento de Ciencias Farmacéuticas: Química Farmacéutica, Facultad de Farmacia, Universidad de Salamanca, CIETUS, IBSAL, 37007 Salamanca, Spain; 6 Departamento de Sanidad Animal, Facultad de Veterinaria, Universidad de León, 12, 24071 León, Spain

**Keywords:** *Trichuris muris*, *Heligmosomoides polygyrus*, Anthelmintic, Benzimidazole, Diamine, Aminoalcohol

## Abstract

**Background:**

Infections by gastrointestinal nematodes cause significant economic losses and disease in both humans and animals worldwide. The discovery of novel anthelmintic drugs is crucial for maintaining control of these parasitic infections.

**Methods:**

For this purpose, the aim of the present study was to evaluate the potential anthelmintic activity of three series of compounds against the gastrointestinal nematodes *Trichuris muris* and *Heligmosomoides polygyrus *in vitro. The compounds tested were derivatives of benzimidazole, lipidic aminoalcohols and diamines. A primary screening was performed to select those compounds with an ability to inhibit *T. muris* L_1_ motility by > 90% at a single concentration of 100 µM; then, their respective IC_50_ values were calculated. Those compounds with IC_50_ < 10 µM were also tested against the adult stage of *T. muris* and *H. polygyrus* at a single concentration of 10 µM.

**Results:**

Of the 41 initial compounds screened, only compounds AO14, BZ6 and BZ12 had IC_50_ values < 10 µM on *T. muris* L_1_ assay, showing IC_50_ values of 3.30, 8.89 and 4.17 µM, respectively. However, only two of them displayed activity against the adult stage of the parasites: BZ12 killed 81% of adults of *T. muris* (IC_50_ of 8.1 µM) and 53% of *H. polygyrus* while BZ6 killed 100% of *H. polygyrus* adults (IC_50_ of 5.3 µM) but only 17% of *T. muris.*

**Conclusions:**

BZ6 and BZ12 could be considered as a starting point for the synthesis of further structurally related compounds.

**Graphical Abstract:**

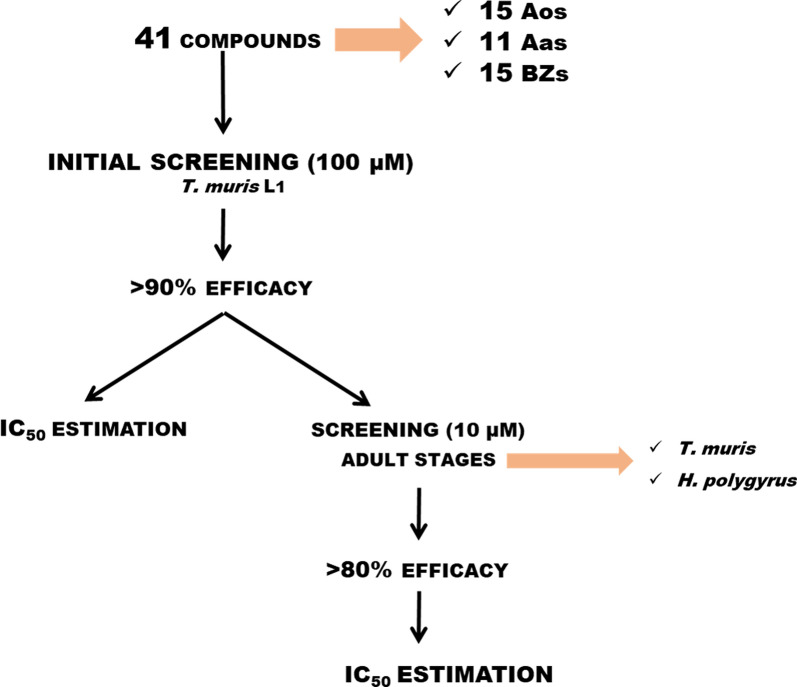

## Background

Soil transmitted helminths (STHs) are a group of human parasitic nematodes that affect around 1.5 billion of the world’s population causing substantial disease and disability [1]. These infections are more common in people living in low- and middle-income countries, in areas with poor access to adequate drinking water, sanitation and hygiene [2]. Of particular importance are infections caused by roundworms (*Ascaris lumbricoides*), whipworms (*Trichuris trichiura*) and hookworms (*Necator americanus* or *Ancylostoma duodenale*) [3]. According to the latest studies, the global incidence of *T. trichiura* infections is estimated to range between 450 million to 1 billion active cases [4].

In animals, the presence of helminth infections has an important economic impact arising directly from reductions in productive yields associated with weight gain, milk production and wool quality [5]. In Europe, the annual economic losses caused by helminth infections in livestock have been estimated at 1.8 billion euros, while those caused only by anthelmintic resistance in the group of gastrointestinal nematodes reach approximately 38 million euros annually [6].

The main control of these infections in humans is based on preventive chemotherapy, employing large-scale administration of anthelmintic drugs to populations at risk. Currently, there are four drugs on the World Health Organization (WHO) model list of essential medicines that are recommended for the treatment of soil-transmitted helminths: albendazole (ABZ), mebendazole (MBZ), levamisole (LEV) and pyrantel pamoate (PYR) [7]. However, the administration of these drugs is not always highly effective, reflecting low cure rates, especially against trichuriasis, when a single oral dose is administered [8, 9]. Moreover, the efficacy of these compounds has decreased significantly over time, showing egg reduction rates dropping from 72.6% in 1995 to 43.3% in 2005 [10].

Additionally, in gastrointestinal nematodes infecting livestock, multiple cases of rapid resistance development have been reported in all continents as a consequence of the abusive use of anthelmintics, mainly benzimidazoles, such as ABZ, and macrocyclic lactones, such as ivermectin (IVM) [11–13].

Considering the spread of anthelmintic resistance in animals and the low efficacy of some benzimidazoles against certain STHs in humans, it is clear that there is an urgent need to search for new drugs to control helminth infections. Therefore, the present study is focused on the determination of the potential in vitro anthelmintic activity of a series of synthetic compounds from different chemical families including benzimidazoles (BZ), lipidic diamines (AA) and aminoalcohols (AO) using two rodent models of gastrointestinal nematodes: *Trichuris muris* and *Heligmosomoides polygyrus* [14, 15]. For this purpose, a total of 41 compounds (15 BZ, 11 AA and 15 AO) were evaluated against two different stages of the parasites, the first stage larvae 1 (L_1_) and adult forms.

## Methods

### Chemical compounds

Compounds belonging to three different chemical families, namely 2-aminoalkan-1-ol (AO), alkane-1,2-diamine (AA) and 2-phenylbenzimidazoles (BZ), were synthesised by previously reported procedures [16–18] (structures summarized in Tables 1, 2 and 3). Stock solutions (10 mM) of these compounds were prepared in 100% dimethyl sulphoxide (DMSO; Sigma-Aldrich^®^) while final dilutions were made with distilled water to maintain a maximum concentration of 1% (v/v) DMSO in the well. All compounds were stored at 4 ºC pending use. To perform all the in vitro assays, levamisole (LEV) at 50 µM was used as positive control (Sigma-Aldrich^®^) and 1% DMSO as negative control.

### Animals and parasites

The complete life cycles of *T. muris* and *H. polygyrus* are maintained at the Swiss Tropical and Public Health Institute (TPH). Experiments were approved by the Swiss national and cantonal authorities (permission no. 2070). Three-week-old female mice (NMRI for *H. polygyrus* and C57BL/6 N for *T. muris*) were allowed to acclimatise to the new environment for 1 week before use. During the acclimatisation period, from day 2 after arrival the animals received 0.25 mg/l dexamethasone (Sigma-Aldrich^®^) in the drinking water to immunosuppress them and facilitate parasite establishment. During the experiment, mice were kept at 22 °C, 50% humidity, with a 12-h light/dark cycle, and water and rodent food (KLIBA NAFAG, Switzerland) were available ad libitum according to Swiss Animal Welfare guidelines. Mice were orally infected with 200 embryonated *T. muris* eggs or 90 *H. polygyrus* L_3_ stage.

### Drug discovery strategy

To investigate the activity against *T. muris*, 41 compounds were first subjected to a primary screening to assess their ability to inhibit *T. muris* motility at the L_1_ stage at a single concentration of 100 µM. Only compounds with the ability to inhibit the motility of > 90% of larvae (activity higher than 90%) progressed to subsequent dose-response evaluation to estimate their half maximal inhibitory concentration (IC_50_) value. Then, those with IC_50_ values < 10 µM were tested on the adult stage of *T. muris* and *H. polygyrus* following a similar protocol as mentioned above: initial screening at 10 µM and estimation of IC_50_ values of those compounds with activities > 80%.

### Evaluation of anthelmintic activity on *T. muris* L_1_s

The assay was performed according to Wimmersberger et al. [19]. Briefly, unembryonated eggs were collected from faeces of infected mice. After storing the eggs for 3 months at room temperature in a dark box, hatching occurred by incubation with 10^7^–10^8^ cells/ml of *Escherichia coli* BL-21 stock. Approximately 40 L_1_ per well was placed into a 96-well plate and incubated for 24 h at 37 ºC and 5% v/v CO_2_ atmosphere, in the presence of 100 μl RPMI-1640 medium supplemented with antibiotic mixture (12.5 μg/ml amphotericin B, 500 U/ml penicillin and 500 μg/ml streptomycin) and 100 μM of the compound to be tested. As a positive control, LEV (Sigma-Aldrich) at a final concentration of 50 μM was used, while wells with medium and 1% DMSO served as negative control. Each compound was tested in duplicate and the assay was repeated on a different day. After 24 h incubation, larval viability was determined by using a binary scale that discriminates alive from dead larvae: “0” = no sign of motion = dead and “1” = motion observed = alive. The percentage of dead larvae was established for each well. To stimulate the movement of all live larvae, 100 µl of hot water (≈ 80 °C) was added to each well following previous protocols [19]. Each larva was observed for 3–5 s. Compounds that showed a killing effect > 90% at 100 μM were selected to determine their IC_50_. For this, L_1_s were incubated with at least six different concentrations of the compound ranging from 100 to 0.41 μM (1:3 serial dilutions).

### Evaluation of anthelmintic activity on *H. polygyrus* and *T. muris* adult worms

The assay was performed according to Karpstein et al. [20]. Briefly, *H. polygyrus* and *T. muris* adults were collected from the caecum (*T. muris*) and colon (*H. polygyrus*) of the mice 2 (*H. polygyrus*) and 7 weeks (*T. muris*) after infection, respectively. All animals were humanely slaughtered by 100% CO_2_ inhalation. Three to four adult worms were placed in each well of a 24-well plate and exposed to the test compounds at a final concentration of 10 μM in RPMI 1640 medium supplemented with 100 U/ml penicillin and 100 μg/ml streptomycin in a final volume of 2.5 ml. *Heligmosomoides polygyrus* medium was supplemented with 12.5 μg/ml amphotericin B and *T. muris* medium with 5% foetal calf serum (iFCS, 100 U/ml). Wells containing 1% (v/v) DMSO in water were included as negative control. Worms were incubated at 37 °C and 5% CO_2_ up to 72 h, after which the drug effect was evaluated using a phenotypic readout. The assay was conducted in duplicate and repeated twice at different days. The condition of the worms was microscopically evaluated according to their phenotype, using a viability scale ranging from 3 to 0 (3: good motility; 2: low motility; 1: very low motility; 0: dead). If adult worms did not move enough for a clear scoring, they were stimulated with 500 μl hot water (≈ 80 °C). Therefore, the effect of the compounds was expressed by the percentage of dead larvae, considering dead those with a score of 0 and alive those with a score ranging from 1 to 3. Compounds with efficacies > 80% at 10 µM were then selected for IC_50_ determination (1:4 serial dilutions ranging from 10 to 0.039 μM).

### Data analysis

IC_50_ values of both assays were calculated based on median effect principle, using the CompuSyn software (CompuSyn, version 3.0.1). These values were defined as the concentration of a drug required to decrease the mean worm’s motility by 50%. The “r” value is the linear correlation coefficient of the median-effect plot; it illustrates the goodness of fit and thus the accuracy of the IC_50_ value.

### Cytotoxicity assays and selectivity indexes

Cytotoxicity assays for most compounds were carried out in a previous study on two different cell lines, the human colorectal adenocarcinoma Caco-2 (ATCC^®^ HTB-37™) and the human hepatocarcinoma HepG2 (ATCC^®^ HB-8065™), using the Alamar Blue staining method, to estimate their toxicity [18, 21]. In the present study, the cytotoxicity of only two compounds, AO14 and AO15, was performed following the protocols mentioned above.

Selectivity indexes (SIs) were calculated by dividing the CC_50_ values obtained in the cytotoxicity assays by the IC_50_ values of the in vitro assays. The greater the SI value, the more selective the compound is in inhibiting *T. muris* activity and the less in inhibiting mammalian cell growth (general cytotoxicity).

## Results

Tables 1, 2 and 3 display the basic scaffold of the different classes of compounds tested and the results of the in vitro assays performed against *T. muris* L_1_ together with cytotoxicity data and SIs. AO and AA compounds are arranged according to the type and size of substituents present on *R*^1^, *R*^2^ and *R*^3^ and to the length (*n*) of the alkylside-chain. BZ compounds are distributed first (R^1^) by the type of substituents at the C-5 (C-6) position of the benzimidazole system and second (*R*^2^) by the substituents on the 2-phenyl ring.

Four AO derivatives (AO5, AO11, AO14 and AO15) displayed activities > 90% in the initial screening at 100 µM against L_1_ *T. muris*, but only AO14 showed an IC_50_ < 10 µM. IC_50_ values of the other three compounds were 25.6, 17.5 and 46.0 µM, respectively (Table 1). For AA derivatives, only compound AA18 reached an activity > 90% (93.55%) in the initial screening showing an IC_50_ value of 21.9 µM (Table 2), while five BZs (BZ1, BZ2, BZ6, BZ12 and BZ13) reached activities > 90% at 100 µM. IC_50_ values in the tested BZs were > 15 µM except for BZ12 and BZ6 with values of 8.89 and 4.17 µM, respectively, against L_1_ *T. muris* (Table 3).

**Table 1:** Base structure and results of L_1_ assays for aminoalcohol (AO) derivatives against *T. muris*

	Compound			*T. muris* L_1_ assay		Cytotoxicity		Selectivity Indexes	
Compound identification	*N*	*R*^1^	*R*^2^	% dead larvae (100 µM)	IC_50_ µM	Caco 2 CC_50_ (µM)	HepG2 CC_50_ (µM)	SI Caco2	SI HepG2
AO1	9	H	H	65.09	–	–	–	–	–
AO2	13	H	H	78.36	–	–	–	–	–
AO3	15	H	H	36.47	–	–	–	–	–
AO4	15	H	Et	85.29	–	–	–	–	–
AO5	17	H	Et	97.04	25.6	13.01 ± 0.27	9.13 ± 0.56	< 1.0	< 1.0
AO6	13	H	Bu	74.44	–	–	–	–	–
AO7	13	H	Hex	53.25	–	–	–	–	–
AO8	13	H	Dec	23.66	–	–	–	–	–
AO9	13	H	Boc	29.63	–	–	–	–	–
AO10	9	Bn	H	61.27	–	–	–	–	–
AO11	13	Bn	H	97.83	17.5	> 20	6.48 ± 0.18	> 1.1	< 1.0
AO12	15	Bn	H	25.55	–	–	–	–	–
AO13	17	Bn	H	27.23	–	–	–	–	–

	Compound		*T. muris* L_1_ assay		Cytotoxicity		Selectivity Indexes	
Compound identification	*N*	*R*^1^	% dead larvae (100 µM)	IC_50_ µM	Caco 2 CC50 (µM)	HepG2 CC50 (µM)	SI Caco2	SI HepG2
AO14	4	H	96.60	3.3	> 15	13.68 ± 1.13	> 4.5	4.1
AO15	6	H	100.00	46.0	32.79 ± 1.28	8.74 ± 1.65	< 1.0	< 1.0

Cytotoxicity data were reported in a previous study [21].

Bn: benzyl; Boc: *tert*-butoxycarbonyl; CC_50_: cytotoxic concentration 50; Dec: *n*-decyl, Et: ethyl; Hex: *n*-hexyl; IC_50_: inhibitory concentration 50; N: length of the alkylside chain; *R*: radical; SI: selectivity index. SI = CC_50_ (Caco-2 or HepG2)/IC_50_

**Table 2:** Base structure and results of L_1_ assays for diamine (AA) derivatives against *T. muris*

	Compound			*T. muris* L_1_ assay		Cytotoxicity		Selectivity indexes	
Compound identification	N	*R*^1^/*R*^2^	*R*^3^	% dead larvae (100 µM)	IC_50_ (µM)	Caco 2 CC_50_ (µM)	HepG2 CC_50_ (µM)	SI Caco2	SI HepG2
AA16	13	H/H	H	0.00	–	–	–	–	–
AA17	17	H/H	H	9.80					
AA18	9	H/H	Boc	93.55	21.9	30.24 ± 1.56	25.20 ± 0.33	1.4	1.1
AA19	13	H/H	Boc	32.47	–	–	–	–	–
AA20	15	H/H	Boc	20.79	–	–	–	–	–
AA21	17	H/H	Boc	23.86	–	–	–	–	–
AA22	13	Et/Et	H	75.02	–	–	–	–	–
AA23	13	H/Et	Boc	87.49	–	–	–	–	–
AA24	13	Et/Et	Boc	61.85	–	–	–	–	–
AA25	13	H/Bu	Boc	28.98	–	–	–	–	–
AA26	13	H/Hex	Boc	12.91	–	–	–	–	–

Cytotoxicity data were reported in a previous study [21].

Bn: benzyl; Boc: *tert*-butoxycarbonyl; Bu: *n*-butyl; CC_50_: cytotoxic concentration 50; Dec: *n*-decyl; Et: ethyl; Hex: *n*-hexyl; IC_50_: inhibitory concentration 50; N: length of the alkylside chain; R: radical; SI: selectivity index. SI = CC_50_ (Caco-2 or HepG2)/IC_50_

**Table 3:** Base structure and results of L_1_ assays for benzimidazole (BZ) derivatives against *T. muris*

	Compound		*T. muris* L_1_ assay		Cytotoxicity		Selectivity Indexes	
Compound identification	*R*^1^	*R*^2^	% dead larvae (100 µM)	IC_50_ (µM)	Caco 2 CC_50_ (µM)	HepG2 CC_50_ (µM)	SI Caco2	SI HepG2
BZ1	Me	4´-OMe	100.00	15.92	11.60 ± 3.42	18.75 ± 1.92	< 1	1.18
BZ2	Me	4´-Cl	91.29	21.94	28.06 ± 1.35	27.11 ± 1.69	1.28	1.23
BZ3	Me	2´,6´-diMe	73.90	–	9.14 ± 2.32	> 25	–	–
BZ4	Me	3´-NO_2_,4´-OMe	40.67	–	9.07 ± 2.35	> 25	–	–
BZ5	Cl	4´-OMe	71.85	–	37.38 ± 3.38	36.03 ± 3.02	–	–
BZ6	Cl	4´-Cl	90.80	4.17	22.58 ± 1.69	17.54 ± 0.91	5.41	4.21
BZ7	Cl	4´- NO_2_	54.74	–	19.14 ± 2.05	21.73 ± 1.65	–	–
BZ8	Cl	2´,6´-diMe	46.82	–	23.28 ± 2.47	30.22 ± 3.35	–	–
BZ9	Cl	3´-NO_2_,4´-OMe	52.43	–	67.31 ± 15.31	53.43 ± 16.61	–	–
BZ10	Cl	3´-NH_2_,4´-OMe	62.06	–	34.70 ± 4.32	44.91 ± 5.94	–	–
BZ11	NO_2_	4´-OMe	53.37		14.37 ± 3.69	22.98 ± 19.57	–	–
BZ12	NO_2_	4´-Cl	95.00	8.89	12.29 ± 1.09	14.64 ± 0.83	1.38	1.65
BZ13	NO_2_	2´,6´-diMe	99.40	18.53	16.78 ± 9.16	> 12.5	< 1	< 1
BZ14	NO_2_	3´-NO_2_,4´-OMe	55.24	–	13.38 ± 3.02	13.64 ± 4.85	–	–
BZ15	NH_2_	4´–OMe	42.26	–	> 25	> 50	–	–

Cytotoxicity data were reported in previous study [19]

CC_50_: cytotoxic concentration 50; IC_50_: inhibitory concentration 50; Me: methyl; *R*: radical; SI: selectivity index. SI = CC_50_ (Caco-2 or HepG2)/IC_50_

Regarding SIs obtained on L_1_ assays, only five compounds belonging to the three families tested (AO14, AA18, BZ2, BZ6 and BZ12) obtained values > 1 on both Caco2 and HepG2 cell lines, with BZ6 reaching the highest SI values, 5.41 in Caco2 cells and 4.21 in HepG2 cells.

The assay performed with AO14, BZ12 and BZ6 against *T. muris* and *H. polygyrus* adults (Table 4) at a fixed concentration of 10 µM showed that BZ12 had an effect of 81% and 53% on *T. muris* and *H. polygyrus* adults after 72 h of exposure, respectively. BZ6 killed 100% of *H. polygyrus* adults at this concentration while it had an activity of 17% against *T. muris*. On the other hand, AO14 did not reach an efficacy > 23% against any parasite. IC_50_ values were calculated on those compounds with activities > 80% at a concentration of 10 µM; therefore, BZ12 showed an IC_50_ value of 8.1 µM on *T. muris* adults while BZ6 showed a lower IC_50_ of 5.3 µM on *H. polygyrus* adults. In the case of the SIs estimated on the adult stage, BZ6 displayed the highest SIs on both cell lines (4.3 in Caco2 and 3.1 in HepG2 cells), while BZ12 showed values closer to one (1.5 in Caco2 and 1.8 in HepG2 cells).

**Table 4:** Results of anthelmintic activity for selected compounds (AO14, BZ12 and BZ6) against *T. muris* and *H. polygyrus* adults at 10 µM

	AO14	BZ6	BZ12
*T. muris* adults			
% Efficacy at 10 µM	21.2	17.2	81.7
IC_50_ (µM)	–	–	8.1
IC_50_ r (µM)	–	–	0.8
SI Caco2	–	–	1.5
SI HepG2	–	–	1.8
*H. polygyrus* adults			
% Efficacy at 10 µM	22.2	100	53.3
IC_50_ (µM)	–	5.3	–
IC_50_ r (µM)	–	0.8	–
SI Caco2	–	4.3	–
SI HepG2	–	3.1	–

IC_50_: inhibitory concentration 50; SI: selectivity index. SI = CC_50_ (Caco-2 or HepG2)/IC_50_

## Discussion

In recent years, the number of new anthelmintics compounds introduced to the market to control the infections produced by gastrointestinal nematodes has been limited, mainly because of economic difficulties in the development and marketing of new drugs [22]. In the last 2 decades, only four drugs have been introduced on the market: emodepside [23], monepantel [24], derquantel [25] and tribendimidine [26]. Therefore, there is a clear need to develop novel anthelmintic drugs for the control of these parasitic worms in humans and farm animals. One of the approaches proposed to alleviate the severe scarcity of anthelmintics is the synthesis of new derivatives of known drugs. Although BZ resistance is present in many gastrointestinal species infecting livestock, the synthesis of novel BZ derivatives may lead to compounds with improved properties such as better solubility and pharmacokinetic profile, resulting in increased effectiveness [27]. Some promising compounds, such as tenvermectin [28], diisopropylphenyl-imidazole [29] and mebendazole hydrochloride [30], have been developed in recent years following this approach.

Based on these assumptions, in the present study, a total of 15 AO and 11 AA derivatives, both structurally related to sphingosine, and 15 benzimidazole derivatives were tested against L_1_ of *T. muris* and adult stages of *T. muris* and *H. polygyrus*. The anthelmintic activity of most of these compounds was previously tested in vitro against the gastrointestinal nematode infecting sheep *Teladorsagia circumcincta* [18, 21] and some of them were also tested against *Leishmania* spp. [31, 32], *Trypanosoma* spp. [17, 33] and *Strongyloides venezuelensis* [34].

The L_1_ assay has proven to be a good tool to screen new potential candidate compounds before carrying out adult motility assays, the in vitro assay of choice, which is more expensive, labour intensive and time-consuming, and it requires the use of live animals [19]. Moreover, the results obtained with the motility assay based on L_1_ seem to correspond to the findings observed with adult *T. muris* [35]. However, some studies showed that L_1_ appears to be more sensitive to drugs than older stages of *T. muris* [36, 37], which can facilitate the discarding compounds with no activity.

In the present study, 10 out of the 41 compounds tested showed activity > 90% against the L_1_ stage of *T. muris* at 100 µM, and only three, namely AO14, BZ12 and BZ6, reached an IC_50_ < 10 µM. The screening performed at a single final concentration of 10 µM on adults showed that only BZ12 and BZ6 had significant activity against the adult stage of *T. muris* and *H. polygyrus*, respectively.

Comparing the results obtained with these derivatives with the previous study carried out against *T. circumcincta* reveals that of the ten compounds screened at 100 µM that showed > 90% activity against *T. muris* L_1_, six (BZ1, BZ2, BZ6, AO11, AO15 and AA18) also showed ovicidal activity against *T. circumcincta*, but only BZ6 reached an IC_50_ value < 10 µM (IC_50_ = 6.54 µM). In the case of *T.* *circumcincta* L_1_, four of them (AO5, AO11, AA18 and BZ6) reached IC_50_ values < 10 µM (IC_50_ for AO5 = 2.87 µM, IC_50_ for AO11 = 1.21 µM, IC_50_ for AA18 = 6.29 µM and IC_50_ for BZ6 = 5.01 µM) and only AO5 and AO11 showed IC_50_ values < 10 µM (IC_50_ for AO5 = 5.55 µM, IC_50_ for AO11 = 4.58 µM) against *T. circumcincta* L_3_. Some of the compounds that did not show activity in the L_1_ *T. muris* assay had shown activity against other parasite models such as *Trypanosoma brucei* (compounds AO4 and AA19 with IC_50_ values close to 0.5 µM) and *Leishmania* spp. (compounds AA25 and AA26). This is also the case for the study carried out on *S. venezuelensis* L_3_, in which compounds AO6, AA18, AA19, AA24 and AA25 showed activity against this nematode (IC_50_ values ranging from 31.9 ± 0.5 μM to 39.1 ± 4.7 μM), but only compound AA18 showed activity against L_1_ of *T. muris* in the current study.

Thus, BZ6 seems to be the only compound reaching IC_50_ values < 10 µM in both eggs and L_1_ of *T. circumcincta* (IC_50_ = 6.54 µM in eggs and IC_50_ = 5.01 µM in L_1_) and also in L_1_ of *T. muris* (IC_50_ = 4.17 µM)*,* with values quite close to each other). However, BZ6 did not have any affect against the adult stage of *T. muris* at a concentration of 10 µM (17.2% of activity), but it was effective against *H. polygyrus* adults (100% of activity) displaying an IC_50_ of 5.3 µM. On the other hand, BZ12 did not produce an effect against any of the stages of *T. circumcincta*, eggs, L_1_ or L_3_, but it showed activity against *T. muris* L_1_ with an IC_50_ of 8.89 µM. Moreover, this BZ12 reached an efficacy of 53.3 and 81.7% on the adult stage of *H. polygyrus* and *T. muris* at 10 µM, respectively, presenting an IC_50_ of 8.1 µM in the latter.

In terms of the relationship between the structure and efficacy of the compounds and focusing on the benzimidazole derivatives, the only group of compounds that has shown significant efficacy on the adult stage of the parasites in this study, we can observe that the presence of a mild basic group such as the NH_2_ group on R_1_ (BZ15) did not induce any measurable effect on the nematode viability, while the combinations of 5-Me–4’-OMe/Cl (BZ1 and BZ2), 5-Cl–4’-Cl (BZ6) and 5-NO_2_–4’-Cl/diMe (BZ12 and BZ13) produced a deadly effect > 90% on the initial screening of *T. muris* L_1_. Regarding the substituent present on the B-phenyl ring (R^2^), 4’-Cl^−^ is required for the anthelmintic effect since all compounds with this substituent at this position showed anthelmintic activity on *T. muris* L_1_ (BZ2, BZ6 and BZ12), including here the two most potent compounds (BZ6 and BZ12), while double substitutions on this ring, such as 3’-NO_2_4’-OMe (BZ4, BZ9 and BZ14) or 3’-NH_2_ 4’-OMe (BZ10), led to inactivity. However, a di-substitution in position 2' and 6' with electron donating groups such as 2',6'-diMe in addition to a polar group in ring A such as 5-NO_2_ (BZ13), gave good anthelmintic inhibitory activity in *T. muris* L_1_ (99.40 inhibition at 100 µM), although its IC_50_ was > 10 µM.

Comparing the results of the adult motility assay of the present study with previous experiments using the marketed human drugs (ABZ, MBZ, LEV and PYR) showed that the IC_50_ values obtained are much lower (8.1 µM for BZ12) since BZ compounds showed a lack of activity on *T. muris* adults and LEV and PYR displayed IC_50_ values around 68 and 57 µM, respectively [19].

All compounds tested against L_1_ had a possible toxic potential, as their SIs were very close to one, except BZ6 and AO14, which reached values > 4 in both cell lines. Regarding the SIs obtained in the adult assays, although they were in any case > 1, BZ6 seems to be a safer candidate than BZ12, as it had SI values of 4.3 for Caco2 cells and 3.1 for HepG2 cells.

## Conclusions

Compounds BZ6 and BZ12 could represent a starting point for the synthesis of further structurally related compounds, as they showed activity against the adult stage of *H. polygyrus* and *T. muris*, respectively.

## Data Availability

The datasets supporting the conclusions of this article are included within the article text and additional files.

## Abbreviations

AA: Diamine
ABZ: Albendazole
ADMET: Absorption, distribution, metabolism, excretion and toxicity
AO: Aminoalcohol
BZ: Benzimidazole
CC_50_: Cytotoxic concentration 50
IC_50_: Inhibitory concentration 50
IVM: Ivermectin
LEV: Levamisole
MBZ: Mebendazole
PYR: Pyrantel pamoate
SI: Selectivity index
